# MLA-ac4C: A N4-Acetylcytidine Site Prediction Model Based on Multi-Layer Attention, Bidirectional GRU, and DenseNet

**DOI:** 10.2174/0113892029407030251229080710

**Published:** 2026-03-19

**Authors:** Jianhua Jia, Guanding Li, Mingwei Sun

**Affiliations:** 1 School of Information Engineering, Jingdezhen Ceramic University, Jingdezhen, 333403, China

**Keywords:** N4-acetylcytidine site prediction, multi-layer attention, Bi-GRU, DenseNet, self-attention mechanism, deep learning

## Abstract

**Introduction:**

N4-acetylcytidine (ac4C) is a highly conserved post-transcriptional modification found in mRNA, known to play a critical role in the regulation of mRNA translation. Traditional experimental approaches for identifying ac4C sites are both time-consuming and costly, underscoring the need for more efficient computational prediction methods. Although various deep learning models have been proposed for ac4C site prediction, most rely on ordinary encoding strategies and simplistic network architectures, which limit their ability to capture high-dimensional features of mRNA sequences. In this study, we propose a novel framework that combines multiple encoding schemes with a hierarchical network structure to improve prediction accuracy.

**Methods:**

We developed a new deep learning model, MLA-ac4C, based on a dual-pathway architecture. In the first pathway, one-hot encoding combined with positional encoding is used to represent the sequence, followed by a Bidirectional Gated Recurrent Unit (Bi-GRU) network to extract global features. In the second pathway, a hybrid encoding scheme integrating nucleotide chemical property (NCP), Electron-Ion Interaction Pseudopotential (EIIP), and Pseudo K-Tuple Nucleotide Composition (PseKNC) is used, and local features are extracted using Densely Connected Convolutional Networks (DenseNet). A multi-layer attention mechanism is then applied to integrate the features from both pathways and capture high-level representations for classification.

**Results:**

Rigorous evaluation on an independent test dataset shows that MLA-ac4C achieves superior performance, with Sensitivity (Sn), Specificity (Sp), Accuracy (Acc), Matthews correlation coefficient (MCC), and Area Under The ROC Curve (AUROC) reaching 83.88%, 85.14%, 84.51%, 69.03%, and 92.74%, respectively. These results outperform those of existing state-of-the-art models.

**Conclusion:**

We present MLA-ac4C, a deep learning model based on Bi-GRU, DenseNet, and a multi-layer attention mechanism. Experimental results demonstrate that it provides a more accurate and efficient approach for predicting ac4C modification sites compared to existing methods.

## 1 INTRODUCTION

RNA plays a central role in gene coding, regulation, and expression, with one of its key characteristics being the presence of numerous chemical modifications that influence its structure and function [1-5]. To date, over 170 types of RNA modifications have been identified [6], predominantly in non-coding RNAs such as tRNA and rRNA, where they are essential for proper transcriptional regulation [7, 8]. Among these modifications, N4-acetylcytidine (ac4C) is a highly conserved post-transcriptional modification catalyzed by N-acetyltransferase 10 (NAT10) [9-11]. Increasing attention has been directed toward ac4C because of its critical role in enhancing both the efficiency and stability of mRNA translation [12-14]. Furthermore, accumulating evidence indicates that ac4C improves the fidelity of tRNA and rRNA translation while also enhancing the thermal stability of tRNA [15-17]. In addition, ac4C modifications are associated with various human diseases, including cancer [18-21]. For instance, Chen *et al.* found that ac4C enhances the invasive and proliferative abilities of Gastric Cancer (GC) cells [22]. These findings highlight the significance of accurately identifying ac4C sites.

Traditionally, ac4C site identification relies on laboratory techniques such as acRIP-seq, developed by Arango *et al.* [23], and Liquid Chromatography-Mass Spectrometry (LC-MS), introduced by Gamage *et al.* [24]. However, these approaches are time-consuming and expensive. Therefore, it is both necessary and timely to explore efficient computational methods, including machine learning and deep learning, for ac4C site prediction [25-29].

In recent years, a wide range of machine learning and deep learning models have been developed for biological sequences site prediction [30-34], and several models have also been specifically applied to the prediction of ac4C sites: Zhao *et al.* proposed PACES, a machine learning model that utilizes Position-Specific Dinucleotide Sequence Profiles (PSDSP) and K-Nucleotide Frequency (KNF), followed by prediction with two random forest classifiers [35]. Alam *et al.* introduced XG-ac4C, which applies various encoding schemes, including EIIP and PseEIIP, and uses XGBoost for classification, resulting in improved accuracy [36]. Wang *et al.* developed DeepAc4C, a deep learning model that incorporates physicochemical features and distributed representations of mRNA sequences, using CNNs to extract high-level features [37]. Su *et al.* presented iRNA-ac4C, which is based on a Gradient Boosting Decision Tree (GBDT) and uses a hybrid encoding involving K-mer, NCP, ANF, and MRMR [38]. Jia *et al.*’s DLC-ac4C integrates C2, NCP, and ND encodings, and employs DenseNet and Bi-LSTM to capture multiscale features with strong generalization capability [39]. Yuan *et al.* proposed DPNN-ac4C, which utilizes position embedding and PseKNC encoding, and incorporates a self-attention mechanism within CNN and Bi-GRU networks, achieving better performance than previous models [40].

Despite their success, existing models generally fail to adequately capture the intra- and inter-dependencies between local and global features. To overcome this limitation, we developed MLA-ac4C, a novel predictor for ac4C site identification that integrates a multi-layer attention mechanism, Bi-GRU [41], and DenseNet [42]. The model is specifically designed to capture correlations between global and local features while effectively learning high-dimensional representations that are most relevant for classification. As a result, MLA-ac4C achieves superior performance, with accuracy and MCC values of 0.8451 and 0.6903, respectively. This demonstrates its ability to identify ac4C sites more efficiently and accurately, thereby not only facilitating early disease detection but also providing a valuable basis for exploring the biological mechanisms underlying ac4C-associated diseases [43-46]. The overall architecture of the model is illustrated in Fig. (**1**).

## 2 MATERIALS AND METHODS

### 2.1 Benchmark Dataset

To ensure a fair comparison with existing models, this study utilizes the same dataset as that used by Su *et al.* [38]. In this dataset, cytidines closest to ac4C peaks are designated as modification sites. Each positive sample consists of a 201-nucleotide sequence centered on a modification site, comprising 100 flanking nucleotides on each side. Negative samples are generated by selecting random cytidines that are not modification sites as the center and constructing 201-nucleotide sequences around them. To reduce sequence redundancy, the CD-HIT tool [47] is employed to remove sequences with similarity thresholds exceeding 0.8 Subsequently, an equal number of negative samples is randomly selected to match the number of positive samples, forming a balanced dataset. The dataset was then randomly split into training and independent test sets at a ratio of 4:1. The final training set contained 2,206 positive samples and 2,206 negative samples, while the independent test set consisted of 552 positive samples and 552 negative samples, as shown in Table (**1**).

### 2.2 Feature Encoding Schemes

An appropriate feature encoding strategy is crucial for the success of a model. Researchers have developed various encoding methods to represent RNA sequences. In this study, we employ five widely used encoding techniques: One-hot encoding, NCP encoding, EIIP encoding, PseKNC encoding, and positional encoding to comprehensively represent RNA sequences from different perspectives.

#### 2.2.1 One-hot Encoding

One-hot encoding is one of the most widely used encoding methods, offering a simple and efficient way to represent RNA sequences. The four nucleotides: adenine (A), uracil (U), cytosine (C), and guanine (G) are encoded as (1, 0, 0, 0), (0, 1, 0, 0), (0, 0, 1, 0), and (0, 0, 0, 1), respectively. Therefore, each sample with a sequence length of *L*=201 is represented as a 201×4 matrix.

#### 2.2.2 NCP Encoding

The Nucleotide Chemical Property (NCP) encoding is based on the chemical structure of nucleotides. It classifies the four nucleotides into two groups according to their ring structure, functional groups, and hydrogen bonding properties, as shown in Table **2** [48]. According to these chemical properties, A, U, C, and G are encoded as (1, 1, 1), (0, 0, 1), (0, 1, 0), and (1, 0, 0), respectively.

#### 2.2.3 EIIP Encoding

The Electron-Ion Interaction Pseudopotential (EIIP) encoding was introduced by Nair *et al.* [49] and was originally used to characterize exon regions in DNA sequences. However, recent studies have shown that EIIP can also effectively represent the electronic properties of RNA sequences [50, 51]. When a sequence is *X*[𝑛] encoded using EIIP, the resulting vector *X*_e_[𝑛] describes the distribution of free electrons along the sequence. Let *X*_e_[𝑘] be the corresponding Discrete Fourier Transform as evaluated by (1).


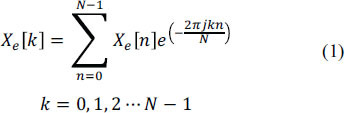


And the corresponding absolute value of the power spectrum 𝑆_𝑒_[𝑘] is (2),


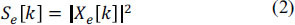


when plotting 𝑆_𝑒_[𝑘] against, the peak values in the generated image correspond to the “hotspot” positions within the sequence. After calculation, the EIIP values for A, U, C, and G are 0.1260, 0.1335, 0.1340, and 0.0806, respectively.

#### 2.2.4 PseKNC Encoding

Pseudo K-tuple Nucleotide Composition (PseKNC) encoding, proposed by Chen *et al.* [52], is designed to characterize the k-mer distribution within biological sequences, enabling the precise extraction of local sequence information [53, 54]. To obtain the PseKNC encoding, the given sequence is first divided into non-overlapping k-mers. The frequency of each distinct k-mer is then calculated to form a frequency vector 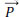
(3).


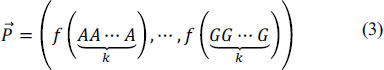


Additionally, pseudo-components are introduced to enhance feature extraction. These pseudo-components include two parts: one derived from the weighting vector for each k-mer frequency (4).


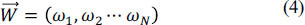


The second component is derived from the correlation vector derived from the relationships among different k-mers (*e.g*., mean and standard deviation) (5).


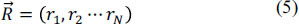


Finally, the PseKNC encoding is obtained by concatenating the frequency vector 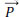, weighting vector 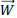, and correlation vector 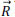
(6).


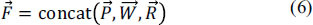


#### 2.2.5 Positional Encoding

Positional encoding, proposed by Vaswani *et al.* [55], is designed to represent the relative or absolute positional information within a sequence. It has been widely adopted in Transformer models and other neural network architectures [56-58]. Positional encoding leverages sine and cosine functions of different frequencies (7):


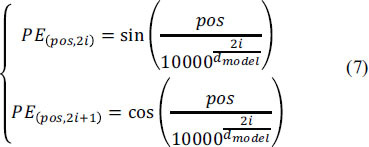


Where 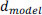 denotes the embedding dimension, represents the specific dimension index, and refers to the position within the sequence. Positional encoding offers the following advantages: each position within a sequence is assigned a unique encoding, and the positional relationships between two elements in a sequence can be derived through affine transformations of their positional encodings (8).


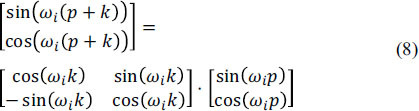


Here 𝑘 represents the offset of the position 𝑝. Based on these two characteristics, this mechanism enables neural networks to capture sequential dependencies more effectively.

### 2.3 Model Architecture

In this study, we propose a model named MLA-ac4C, which consists of two main paths. In the first path, RNA sequences are first encoded using One-hot encoding, supplemented with positional encoding. This encoded representation is then processed using a Bi-GRU network, which extracts global features and long-range dependencies from the sequence. To further enhance feature correlations and emphasize crucial information, we incorporate a multi-head self-attention mechanism. In the second path, we employ a hybrid encoding scheme that integrates three feature representations: NCP encoding, EIIP encoding, and PseKNC encoding to comprehensively capture the physicochemical properties of RNA sequences. The resulting feature matrix is then processed by a DenseNet, which effectively extracts local features while preserving essential raw information. Subsequently, a multi-head self-attention mechanism is applied to highlight significant features. Finally, the high-dimensional global features extracted from the first path and the high-dimensional local features obtained from the second path are concatenated. A final multi-head self-attention module, termed Multi-Layer Attention, is then applied to further refine the feature representation by simultaneously incorporating both global and local information. The final feature vector is subsequently fed into an MLP for classification. The architecture of the model is illustrated in Fig. (**1**).

#### 2.3.1 Bi-GRU

The Bidirectional Gated Recurrent Unit (Bi-GRU) is a type of Recurrent Neural Network (RNN) [59-61] designed for extracting sequential features. Compared to standard RNNs, Bi-GRU employs two independent GRU layers operating in opposite directions, allowing it to capture both past and future contextual information at each time step. GRU, a widely used RNN variant, efficiently captures long-range dependencies while utilizing fewer parameters than Long Short-Term Memory (LSTM) [62] networks. Additionally, GRU helps alleviate the issues of vanishing and exploding gradients, making it more computationally efficient. The structure of GRU is illustrated in Fig. (**2**).

Bi-GRU processes an input sequence by maintaining a forward hidden state and a backward hidden state . The final hidden representation at each time step is computed as follows (9-15):


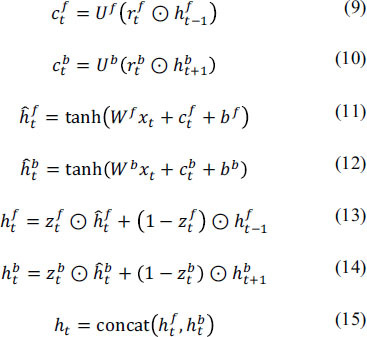


Where 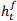 and 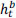 are the outputs of the forward and backward GRU layers, respectively. 𝑊 and 𝑈 are weight matrices, 𝑏 represents bias terms, ⊙ indicates element-wise multiplication, tanh(.) represents the hyperbolic tangent activation function. The update gate (𝑧_𝑡_) and reset gate (𝑟_𝑡_) for both directions are computed as follows (16-19):


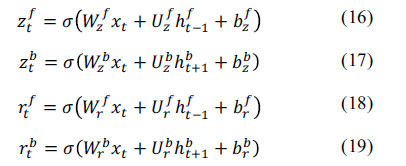


Where 𝜎(.) denotes the sigmoid activation function, which maps inputs into the interval (0, 1), The update gate 𝑧_𝑡_ regulates the extent to which the previous hidden state is retained. When 𝑧_𝑡_ approaches 1, the model retains more past information; when it approaches 0, more information is discarded. By adjusting the gate values in both forward and backward directions, Bi-GRU effectively controls information flow, enabling the capture of long-range dependencies within sequential data.

#### 2.3.2 DenseNet

DenseNet, an improved variant of Convolutional Neural Networks (CNNs), was proposed by Huang *et al.* [42] to address the vanishing gradient problem, enhance feature reuse, and significantly reduce the number of parameters. Unlike ResNet, introduced by He *et al.* [63], which employs residual connections between adjacent layers, DenseNet establishes dense connections between all layers. Specifically, each layer receives as input the concatenation of feature maps from all preceding layers, as formulated below (20):


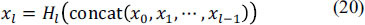


Where x_l_ represents the output of the l-th layer, H_l_(.) denotes a composite function consisting of convolution operations, batch normalization, ReLU activation, and maxpooling. This dense connectivity pattern facilitates more efficient gradient propagation, encourages feature reuse, and reduces redundancy, ultimately leading to a more compact and computationally efficient model. The structure of DenseNet is illustrated in Fig. (**3**).

#### 2.3.3 Multi-layer Self-attention

The self-attention mechanism, proposed by Vaswani *et al.* [55], is inspired by human attention mechanisms and employs a Query-Key-Value (QKV) representation to compute attention scores across a sequence. Additionally, the multi-head self-attention mechanism utilizes multiple independent linear transformations, referred to as attention heads, to project the input into different attention subspaces. Each head independently computes attention scores, allowing the model to capture sequence features from multiple perspectives. Unlike convolutional layers and recurrent networks, self-attention is not constrained by a receptive field or sequential dependencies, making it highly effective in modeling long-range dependencies. Consequently, it has become a fundamental component in modern deep learning architectures [64-69].

In this study, we employ Multi-layer self-attention to effectively integrate features extracted from both pathways of our model. Specifically, we first apply self-attention independently to global features extracted from the Bi-GRU network and local features extracted from the DenseNet network. This step enables the model to capture contextual dependencies within each pathway while highlighting salient features. Subsequently, the outputs from both pathways are concatenated and processed by an additional self-attention layer, which extracts cross-pathway relationships. By leveraging this hierarchical attention mechanism, our model effectively captures both intra-pathway and inter-pathway dependencies, leading to a more comprehensive and discriminative feature representation. The formula for the Multi-layer self-attention is represented as follows (21):


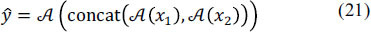


Where 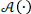 denotes the application of a multi-head self-attention mechanism, 𝑥_1_ and 𝑥_2_ represent the outputs of the two respective network pathways. The multi-head self-attention mechanism is computed using the following equations (22-24):


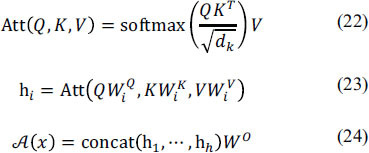


Where Q, K and V represent the query, key, and value matrices, respectively, all of which are derived from different linear transformations of the input sequence. ℎ_𝑖_ is a different attention head. 𝑑_𝑘_ is the dimension of each attention head. 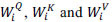 are learnable projection matrices of the 𝑖-th head. 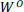 is the output projection matrix. By applying multiple attention heads in parallel, the multi-head self-attention mechanism enables the model to capture diverse feature representations from different subspaces, thereby enhancing its ability to learn complex relationships within the sequence. The structure of the self-attention mechanism is illustrated in Fig. (**4**).

#### 2.3.4 Performance Evaluation

To comprehensively and accurately evaluate the performance of the model, we employ five commonly used evaluation metrics, including accuracy (Acc), sensitivity (Sn), specificity (Sp), Matthews correlation coefficient (MCC), and the Area Under The Receiver Operating Characteristic Curve (AUROC). These metrics are computed using the following formulas (25-28):


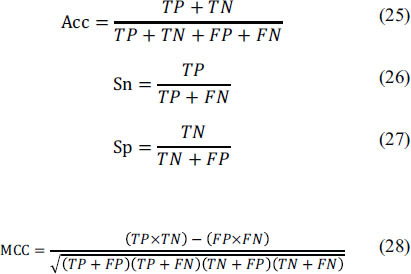


Where 𝑇𝑃 (True Positives) represents the number of correctly predicted positive samples. 𝑇𝑁 (True Negatives) represents the number of correctly predicted negative samples. 𝐹𝑃 (False Positives) represents the number of negative samples incorrectly predicted as positive. 𝐹𝑁 (False Negatives) represents the number of positive samples incorrectly predicted as negative. Additionally, the AUROC value corresponds to the area under the ROC curve, where a higher value indicates better classification performance.

## 3 RESULTS AND DISCUSSION

### 3.1 Comparison of Different Encoding Methods

In this study, we employed a combination of three encoding methods, NCP, EIIP, and PseKNC, in the second pathway of our network to comprehensively capture the physicochemical characteristics of RNA sequences. To systematically evaluate the impact of different encoding combinations on model performance, we conducted an ablation study on all seven possible combinations of these three encoding schemes. Importantly, all other model parameters were kept constant during the experiments to ensure fair comparison. The results obtained on the independent test set are summarized in Table (**3**).

The results indicate that using NCP encoding alone yields the highest Sp of 0.8967; however, it also results in the lowest Sn of 0.7717. This suggests that NCP alone tends to bias the model toward negative class predictions. Given that our dataset is balanced, it is essential to maintain a reasonable trade-off between sensitivity and specificity to improve overall classification accuracy. The combination of NCP and PseKNC substantially improves both sensitivity and accuracy, achieving the highest Sn of 0.8569 and a second-highest Acc of 0.8433, indicating that the integration of complementary feature representations enhances model performance. However, the Sp for this combination drops to 0.8297, suggesting a slight tendency toward positive class predictions. Finally, the combination of all three encodings, NCP, EIIP, and PseKNC, achieves the best overall performance, with the highest Acc of 0.8451, the highest MCC of 0.6903, and the highest AUC of 0.9274. This configuration effectively balances classification performance across both classes while avoiding the introduction of excessive computational complexity. Therefore, we select the NCP+EIIP+PseKNC combination as the preferred encoding scheme for our model.

### 3.2 Comparison of Different Numbers of Dense Blocks

To investigate the impact of different numbers of dense blocks on model performance, we conducted an ablation study on the independent test set while keeping all other parameters unchanged. The results are illustrated in Fig. (**5a**). As observed, when the number of dense blocks is fewer than three, Acc and MCC increase with the addition of more dense blocks. Notably, setting the number of dense blocks to zero indicates the replacement of DenseNet with a simple CNN. This trend suggests that DenseNet contributes to the extraction of higher-dimensional features. However, when the number of dense blocks exceeds three, the improvements in Acc and MCC become marginal, possibly due to limitations in feature representation or the network architecture, which may prevent the extraction of more complex features. Furthermore, increasing the number of dense blocks leads to a significant rise in model parameters and computational cost. Considering both model performance and complexity, we selected three dense blocks as the optimal configuration.

### 3.3 Importance of Multi-layer Attention

In this study, we employed a multi-layer attention mechanism to perform hierarchical feature extraction from two distinct network pathways. To evaluate its effectiveness, we conducted ablation experiments comparing three configurations: a model without attention, a model with single-layer attention, and a model with multi-layer attention. The performance differences among these configurations are illustrated in Fig. (**5b**). The results demonstrate that the model incorporating multi-layer attention consistently outperforms both the single-layer attention model and the version without attention across all evaluation metrics. Specifically, compared to the single-layer attention model, the multi-layer attention configuration yields improvements of 2.26%, 0.45%, 1.36%, 2.64%, and 1.32% in Sn, Sp, Acc, MCC, and AUC, respectively. These results indicate that the multi-layer attention mechanism significantly enhances model performance by more effectively capturing important sequence features.

Furthermore, we visualized the high-dimensional representations learned through single-layer and multi-layer attention using t-distributed stochastic neighbor embedding (t-SNE) [70-72], as shown in Fig. (**5c**). (t-SNE is a nonlinear dimensionality reduction technique widely used to visualize high-dimensional data in a low-dimensional space). The t-SNE plots clearly show that the features derived from multi-layer attention result in a more distinct separation between positive and negative samples. This suggests that multi-layer attention effectively integrates global and local information from the two network branches, leading to the learning of more discriminative high-level representations and, consequently, improved classification performance.

### 3.4 Comparison with Existing Models

To comprehensively evaluate the performance of MLA-ac4C, we compared it against six existing models, PACES [35], XG-ac4C [36], DeepAc4C [37], iRNA-ac4C [38], DLC-ac4C [39], and DPNN-ac4C [40], using both 10-fold cross-validation and an independent test set, as shown in (Tables **4** and **5**). Among them, the performance metrics for DLC-ac4C were obtained from the study proposed by Jia *et al.* [39], while those for the remaining five models were sourced from the work of Yuan *et al.* [40].

As presented in Table **4**, MLA-ac4C achieves the highest values in four evaluation metrics during 10-fold cross-validation: Sp of 0.8576, Acc of 0.8490, MCC of 0.6978, and AUC of 0.9346. Compared to the most recent model, DPNN-ac4C, these values represent respective improvements of 0.15%, 1.01%, 4.23%, and 0.74%. While MLA-ac4C's Sn is lower than that of XG-ac4C (0.9336), the latter exhibits a strong bias toward the positive class, as indicated by its low Sp of only 0.5462. Excluding XG-ac4C, MLA-ac4C attains the highest Sn value of 0.8422, outperforming DPNN-ac4C by 2.04%, thereby demonstrating the superior performance of our proposed model.

To further validate the robustness of MLA-ac4C, we also conducted evaluations on an independent test set, as shown in Table **5**, and visualized the results using radar charts and ROC curves in Fig. (**6a**) and Fig. (**6b**). Consistent with the cross-validation results, MLA-ac4C achieves the best performance in all metrics except Sn. Although its Sn is lower than that of XG-ac4C and DLC-ac4C, MLA-ac4C shows significantly higher Sp, 31.60% and 5.43% higher than XG-ac4C and DLC-ac4C, respectively, indicating a more balanced classification of both positive and negative samples. Consequently, MLA-ac4C achieves higher Acc and MCC scores. When compared to DPNN-ac4C, MLA-ac4C delivers all five evaluation metrics’ improvements of 2.10% in Sn, 0.36% in Sp, 1.73% in Acc, 3.25% in MCC, and 1.71% in AUC, clearly demonstrating that MLA-ac4C outperforms existing models and provides more accurate identification of ac4C sites.

### 3.5 Motif Analysis

To investigate potential sequence motifs surrounding ac4C modification sites and to explore possible biological implications, we performed motif analysis on all positive samples using the STREME tool [73]. As shown in Fig. (**6c**), a highly significant enrichment of cytosine was observed at specific positions near the ac4C sites. This finding is consistent with the observations reported by Arango *et al.* [12], Yuan *et al.* [40], and Su *et al.* [38], who also noted a higher cytosine frequency in sequences containing ac4C modifications compared to non-acetylated mRNA transcripts. One plausible biological explanation for this enrichment is that cytosine-rich sequences may facilitate interactions between mRNA and homologous tRNA, thereby enhancing the translation efficiency of transcripts harboring ac4C modifications [74]. In addition to this motif, other identified motifs are provided in the Supplementary Materials section. We hope that these findings will contribute to a deeper understanding of the biological roles of ac4C modification sites.

## CONCLUSION

In this study, we propose a novel predictive model for ac4C site identification, termed MLA-ac4C. The model employs a dual-pathway architecture combined with a multi-head self-attention mechanism to effectively capture both intra- and inter-dependencies between global and local sequence features. Experimental results demonstrate that MLA-ac4C achieves superior predictive performance on the independent test set, with sensitivity (Sn), specificity (Sp), accuracy (Acc), Matthews correlation coefficient (MCC), and area under the ROC curve (AUROC) reaching 83.88%, 85.14%, 84.51%, 69.03%, and 92.74%, respectively. These results consistently outperform state-of-the-art models across all five evaluation metrics.

Despite its strong performance, MLA-ac4C still has certain limitations. First, the dataset utilized in this study is relatively small, which may limit the model’s capacity to learn more complex, high-dimensional representations through deep learning [75, 76]. Second, we did not conduct a comprehensive evaluation of all possible RNA encoding schemes, and there may exist more optimal combinations than those explored here [77, 78]. In future work, we plan to investigate adaptive encoding strategies and leverage larger, more diverse datasets to further improve the robustness and generalizability of the model.

## Figures and Tables

**Fig. (1):**
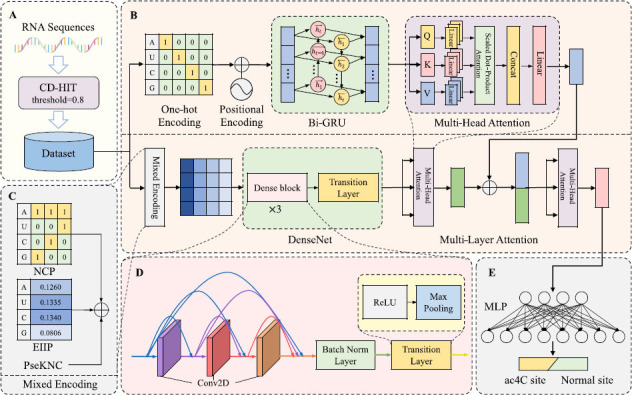
The overall structure of the model. (**A**) Data collection and preprocessing. (**B**) Main structure of the model. (**C**) The mixed encoding module. (**D**) The structure of the Dense block. (**E**) Classification module.

**Fig. (2):**
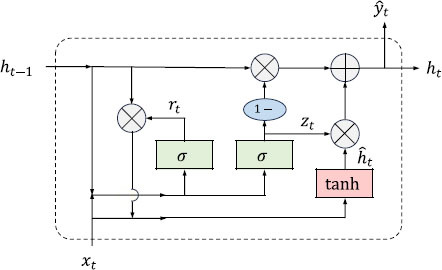
The structure of GRU. 𝜎 denotes the sigmoid activation function.

**Fig. (3):**
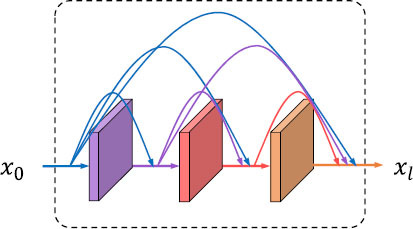
The structure of DenseNet, curved arrows between different layers represent residual connections.

**Fig. (4):**
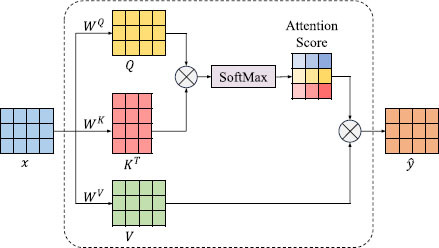
The structure of self-attention mechanism.

**Fig. (5):**
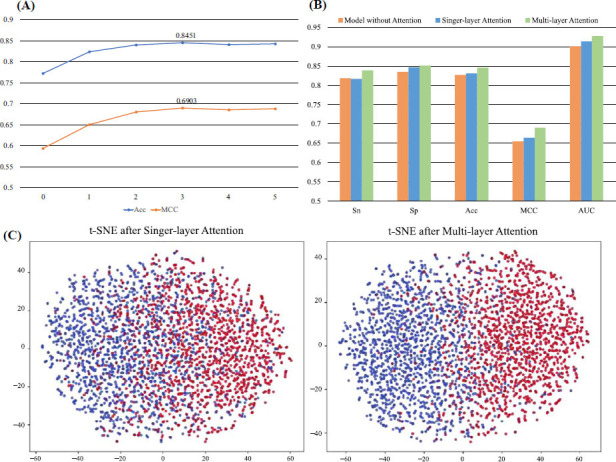
(**A**) Comparison of performance of models with different numbers of dense blocks on independent test sets. (**B**). Comparison of performance of models with different attention module on independent test sets. (**C**). The t-SNE plots of different attention module on the 10-fold cross-validation of the training set.

**Fig. (6):**
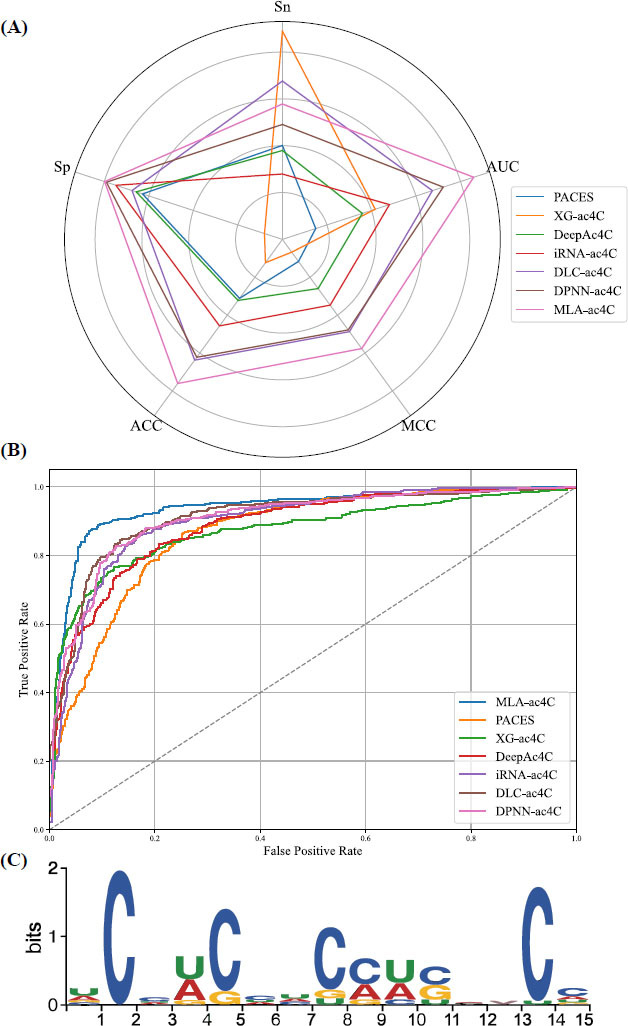
(**A**) The radar charts of different models’ performance on independent test sets. (**B**). The ROC curves of different models on independent test sets. (**C**). A motif that exists in positive sample sequences.

**Table 1:** Distribution of benchmark datasets.

**-**	**Training Set**	**Test Set**	**Total**
Positive	2206	552	2758
Negative	2206	552	2758
Total	4412	1104	5516

**Table 2:** Chemical properties of different nucleotides.

**Chemical Property**	**Class**	**Nucleotides**
Ring structure	Purine	A, G
	Pyrimidine	U, C
Functional groups	Amino	A, C
	Keto	U, G
Hydrogen bond	Strong	C, G
	Weak	A, U

**Table 3:** Ablation results for different encoding methods on the independent test set. (The best results for each metric are highlighted in bold).

**-**	**Sn**	**Sp**	**Acc**	**MCC**	**AUC**
NCP	0.7717	**0.8967**	0.8342	0.6738	0.9123
EIIP	0.7971	0.8678	0.8324	0.6665	0.9133
PseKNC	0.8152	0.8551	0.8351	0.6708	0.9118
NCP+EIIP	0.7754	0.8949	0.8350	0.6751	0.9172
NCP+PseKNC	**0.8569**	0.8297	0.8433	0.6868	0.9126
EIIP+PseKNC	0.8152	0.8641	0.8397	0.6802	0.9144
NCP+EIIP+PseKNC	0.8388	0.8514	**0.8451**	**0.6903**	**0.9274**

**Table 4:** Comparison of different network architecture models on the 10-fold cross-validation of the training set. (The best results for each metric are highlighted in bold.).

**-**	**Sn**	**Sp**	**Acc**	**MCC**	**AUC**
PACES	0.7849	0.7702	0.7856	0.5435	0.8438
XG-ac4C	**0.9336**	0.5462	0.7407	0.5223	0.8331
DeepAc4C	0.8062	0.8056	0.8026	0.6032	0.8641
iRNA-ac4C	0.7702	0.8301	0.8003	0.6010	0.8750
DLC-ac4C	0.8319	0.7726	0.8007	0.6064	0.8774
DPNN-ac4C	0.8218	0.8561	0.8389	0.6655	0.9272
MLA-ac4C	0.8422	**0.8576**	**0.8490**	**0.6978**	**0.9346**

**Table 5:** Comparison of different network architecture models on the independent test set. (The best results for each metric are highlighted in bold.).

**-**	**Sn**	**Sp**	**Acc**	**MCC**	**AUC**
PACES	0.7963	0.7765	0.7889	0.5385	0.8387
XG-ac4C	**0.9135**	0.5354	0.7654	0.5223	0.8721
DeepAc4C	0.7911	0.7889	0.7903	0.5857	0.8649
iRNA-ac4C	0.7670	0.8291	0.8071	0.6146	0.8801
DLC-ac4C	0.8623	0.7971	0.8297	0.6608	0.9042
DPNN-ac4C	0.8178	0.8478	0.8278	0.6578	0.9103
MLA-ac4C	0.8388	**0.8514**	**0.8451**	**0.6903**	**0.9274**

## Data Availability

The dataset and source code for MLA-ac4C are freely available on https://github.com/LIguanding17/MLA-ac4C. Additionally, the online ac4C site prediction using our model can be accessed *via* https://mla-ac4c-rvzgyquwvi84wxgpgzmrwv.streamlit.app/.

## LIST OF ABBREVIATIONS

ac4C: N4-acetylcytidine
Acc: Accuracy
AUROC/AUC: Area Under the ROC Curve
Bi-GRU: Bidirectional Gated Recurrent Unit
CNN: Convolutional Neural Network
DenseNet: Densely Connected Convolutional Network
EIIP: Electron-Ion Interaction Pseudopotential
LSTM: Long Short-Term Memory
MCC: Matthews Correlation Coefficient
MLP: Multi-Layer Perceptron
NAT10: N-acetyltransferase 10
NCP: Nucleotide Chemical Property
PseKNC: Pseudo K-tuple Nucleotide Composition
RNN: Recurrent Neural Network
Sn: Sensitivity
Sp: Specificity
t-SNE: t-distributed Stochastic Neighbor Embedding
