# Supplementary material for: MLA-ac4C: A N4-Acetylcytidine Site Prediction Model Based on Multi-Layer Attention, Bidirectional GRU, and DenseNet
Source: Curr Genomics. 2026 Mar 19;26(6):713–25. doi: 10.2174/0113892029407030251229080710 (PMC13154229; doi:10.2174/0113892029407030251229080710)
Supplement: Supplementary file 1 [file CG-26-6-713_SD1.pdf]

Supplementary Material

MLA-ac4C: A N4-Acetylcytidine Site Prediction Model Based on Multi-Layer Attention, Bidirectional GRU, and DenseNet

Jianhua Jia[1,*], Guanding Li[1] and Mingwei Sun[1]

[1]*School of Information Engineering, Jingdezhen Ceramic University, Jingdezhen, 333403, China*

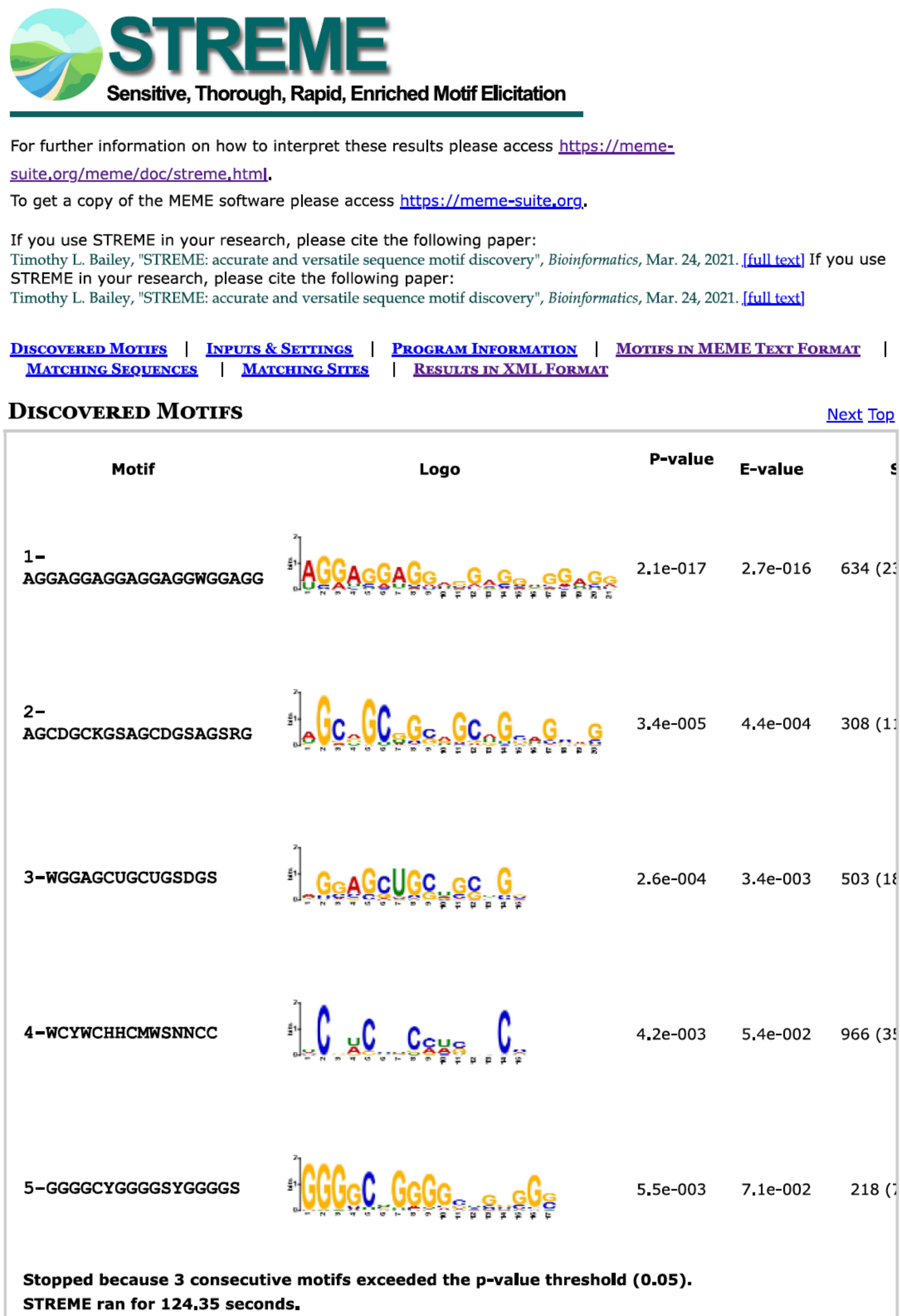

For further information on how to interpret these results please access https://meme-suite.org/meme/doc/streme.html.
To get a copy of the MEME software please access https://meme-suite.org.

If you use STREME in your research, please cite the following paper:
Timothy L. Bailey, "STREME: accurate and versatile sequence motif discovery", *Bioinformatics*, Mar. 24, 2021. [full text] If you use STREME in your research, please cite the following paper:
Timothy L. Bailey, "STREME: accurate and versatile sequence motif discovery", *Bioinformatics*, Mar. 24, 2021. [full text]

DISCOVERED MOTIFS | INPUTS & SETTINGS | PROGRAM INFORMATION | MOTIFS IN MEME TEXT FORMAT | MATCHING SEQUENCES | MATCHING SITES | RESULTS IN XML FORMAT

DISCOVERED MOTIFS Next Top

Motif	Logo	P-value	E-value	S
1-AGGAGGAGGAGGAGGWGGAGG		2.1e-017	2.7e-016	634 (2
2-AGCDGCKGSAGCDGSAGSRG		3.4e-005	4.4e-004	308 (1
3-WGGAGCUGCUGSDGS		2.6e-004	3.4e-003	503 (1
4-WCYWCHHCMWSNNCC		4.2e-003	5.4e-002	966 (3
5-GGGGCYGGGGSYGGGGS		5.5e-003	7.1e-002	218 (

Stopped because 3 consecutive motifs exceeded the p-value threshold (0.05).
STREME ran for 124.35 seconds.

Motif	Logo	P-value	E-value	S
6-CGGCCCCGGCCCCCB	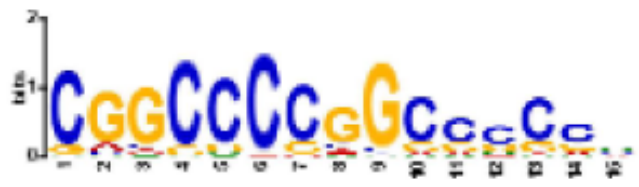	9.5e-003	1.2e-001	272 (9
7-GCCGCSGCSGCCGCSGCSG	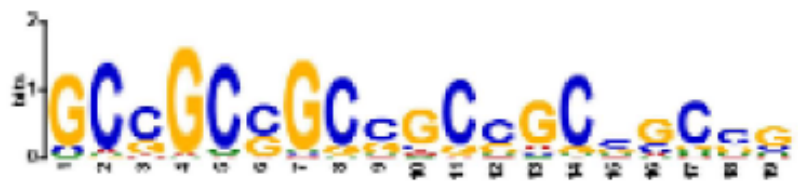	2.3e-002	3.0e-001	393 (14
8-GGCGGGGAGG	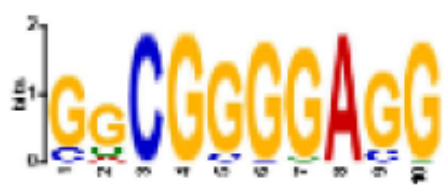	3.4e-002	4.4e-001	59 (2
9-AGGAAGAGGAGGAAG	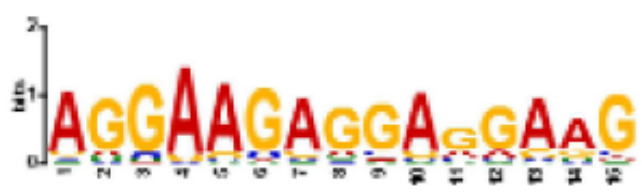	3.4e-002	4.4e-001	40 (1
10-AAGCACAA	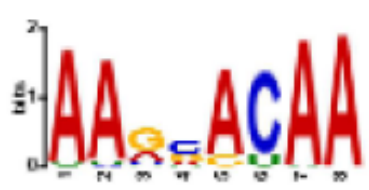	3.6e-002	4.7e-001	117 (4
11-GACUUUGAC	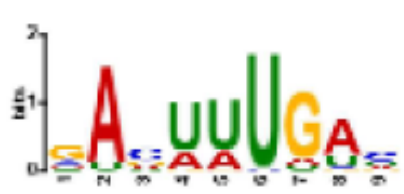	3.9e-001	5.1e+000	350 (12
12-GGCCSUGGSCVUGGMSG	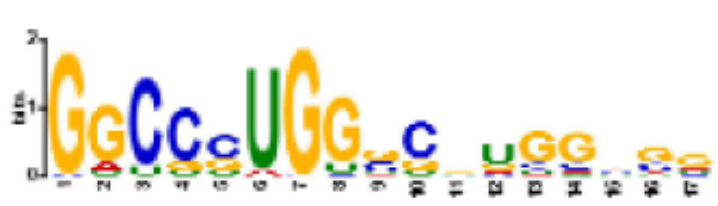	5.0e-001	6.5e+000	195 (7
13-AAUAACAU	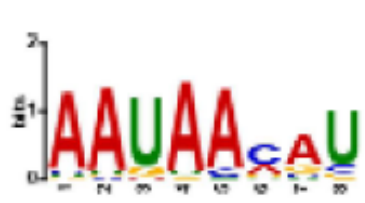	6.4e-001	8.3e+000	90 (3

Stopped because 3 consecutive motifs exceeded the p-value threshold (0.05).
STREME ran for 124.35 seconds.

Inputs & Settings

Previous Next Top

Sequences

Role	Source	Alphabet	Sequence Count	Total Size
Positive (primary) Sequences	.fasta	RNA	2758	554358
Negative (control) Sequences	2-Order Shuffled Positive Sequences	RNA	2758	554358

Background Model

Source: built from the negative (control) sequences

Source: built from the negative (control) sequences

Order: 2 (only order-0 shown)

	Name	Freq.	Bg.
A	Adenine	0.183	0.183
C	Cytosine	0.313	0.313
G	Guanine	0.349	0.349
U	Uracil	0.155	0.155

	Name	Freq.	Bg.
A	Adenine	0.183	0.183
C	Cytosine	0.313	0.313
G	Guanine	0.349	0.349
U	Uracil	0.155	0.155

Other Settings

**Strand Handling**	This alphabet only has one strand.
**Objective Function**	Differential Enrichment
**Statistical Test**	Fisher Exact Test
**Minimum Motif Width**	8
**Maximum Motif Width**	21
**Sequence Shuffling**	Negative sequences are positives shuffled preserving 3-mer frequencies.
**Test Set**	10% of the input sequences were randomly assigned to the test set.
**Word Evaluation**	Up to 25 words of each width from 8 to 21 were evaluated to find seeds.
**Seed Refinement**	Up to 4 seeds of each width from 8 to 21 were further refined.
**Refinement Iterations**	Up to 20 iterations were allowed when refining a seed.
**Random Number Seed**	0
**Trimming of Control Sequences**	Trimming of control sequences was allowed.
**Total Length**	The total length of each sequence set was limited to 4.00e+6.
**Maximum Motif p-value**	Stop when the p-value is greater than 0.05 for 3 consecutive motifs.
**Maximum Motifs to Find**	No maximum number of motifs.
**Maximum Run Time**	14400 seconds.

Previous Top

STREME version
5.5.7 (Release date: Wed Jun 19 13:59:04 2024 -0700)

Reference
Timothy L. Bailey, "STREME: accurate and versatile sequence motif discovery", *Bioinformatics*, Mar. 24, 2021. [full text]

Reference

Timothy L. Bailey, "STREME: accurate and versatile sequence motif discovery", *Bioinformatics*, Mar. 24, 2021. [full text]

Command line

```
streme --verbosity 1 --oc . --rna --totallength 4000000 --time 14400 --minw 8 --maxw 21 --thresh
0.05 --align center --p .fasta
```

高速下载
